# Understanding human evolutionary history: a meeting report of the Swedish Royal Academy of Sciences symposium of modern human genetic variation

**DOI:** 10.1186/2041-2223-3-7

**Published:** 2012-03-24

**Authors:** Joshua M Akey

**Affiliations:** 1 Department of Genome Sciences, University of Washington, Foege Building S-303 1705 NE Pacific, Seattle, WA 98195-5065, USA

**Keywords:** Human evolution, Archaic admixture, Selection, Demography, Complex traits

## Abstract

A report on the Swedish Royal Academy of Sciences Symposium on Modern Human Genetic Variation. Stockholm, Sweden. January 19-20, 2012.

## 

The story of anatomically modern humans has captivated the imagination of nearly everyone who has ever wondered about our past, our relationship with one another and with other species, and how our evolutionary history has shaped extant patterns of genetic variation and phenotypic diversity. To highlight recent progress and future avenues of research in human evolutionary history, the Swedish Royal Academy of Sciences recently convened a Symposium on Modern Human Genetic Variation, which featured 12 invited speakers. The two-day Symposium was open to the public, and the large audience it attracted underscores the intense interest of both scientists and the public-at-large in studies of human evolutionary history. Two striking themes permeated this Symposium. First, many of the talks discussed the use of next-generation sequencing data to delineate patterns of human genomic diversity. It is remarkable how transformative this technology has become in such a small amount of time. Second, it was clear that comprehensive insights into human evolutionary history requires interdisciplinary approaches, and the talks were noteworthy in the diversity of approaches and topics that were being pursued to further illuminate the complex interaction of evolutionary, cultural and demographic forces that have shaped the evolutionary trajectory of humans. Below, I summarize some of the major topics and insights that emerged over the course of this Symposium.

## Comprehensively cataloging human genomic variation

Despite decades of research, the catalog of human variation remains incomplete because of the limited number of worldwide populations that have been studied, inability to obtain genome-scale sequencing data in large numbers of individuals, and difficulty in comprehensively accessing complex types of variation, such as copy number variants (CNVs). A better understanding of the full spectrum of human genomic diversity is critical for delineating our evolutionary past and the heritable basis of phenotypic variation and disease susceptibility. For example, Stephan Schuster (Pennsylvania State University, University Park, USA) presented whole-genome sequence data from several individuals of South African ancestry, and over one million novel variants were discovered. In light of the large number of (primarily non-African) individuals that have been sequenced to date, these data are striking and clearly demonstrate that a considerable number of variants have yet to be discovered, particularly in traditionally underrepresented populations.

Lars Feuk (Uppsala University, Sweden) showed that changes in DNA sequence do not provide a complete portrait of human genetic diversity, and differences in DNA content (broadly defined as CNVs and inversions) among genomes is a significant source of genetic variation. By careful analysis of the breakpoints for many structural variants, general insights into the mechanistic basis of such polymorphisms were obtained. For example, Feuk demonstrated that replication based mechanisms could explain most small structural variants, whereas non-allelic homologous recombination disproportionately contributed to CNVs > 10 kb and inversion polymorphisms. The application of next-generation sequencing technology to large and globally diverse population samples is poised to provide increasingly accurate and complete collections of extant patterns of genomic variation.

## The role of demographic history and selection in shaping patterns of human genetic diversity

The primary forces shaping patterns of DNA sequence variation are natural selection and genetic drift (that is, the stochastic change of allele and haplotype frequencies) mediated by demographic history. Thus, a considerable amount of work has focused on identifying targets of selection and estimating demographic parameters of human history, such as levels of population structure and the times and magnitudes of changes in population size. To this end, a number of talks focused on recent insights into how selection and demography have influenced human history. For instance, Sohini Ramachandran (Brown University, Providence, RI, USA) described her work on a serial founder effect model, which generates testable predictions about patterns of diversity among populations that have dispersed over long distances. Ramachandran demonstrated that a serial founder effect model of modern humans that originated in Africa explains many features of human genetic variation in both geographically diverse populations and in the peopling of the Americas. Moreover, Noah Rosenberg (Stanford University, Stanford, CA, USA) showed the serial founder effect model could also account for patterns of homozygosity and linkage disequilibrium observed in worldwide populations. Rosenberg also presented results on detailed analyses of imputation, a major finding of which was that imputation accuracy increases when appropriate reference samples for a given population are used. These results are of considerable practical importance, and reinforce the message from Schuster that a better sampling of human diversity is critically needed.

In another talk focused on demography, Mattias Jakobsson (Uppsala University, Sweden) presented novel data on the impact of the agricultural revolution on the genetics of contemporary European populations. Specifically, Jakobsson and colleagues obtained nearly 250 Mb of sequence from three 5,000-year-old remains of Neolithic hunter-gatherers and one Neolithic farmer excavated in Scandinavia. Analysis of these sequences in the context of the present day European gene pool suggests that the spread of agriculture involved the northward migrations of farmers. Thus, these data provide the most direct and compelling support for the demic diffusion model of agriculture (as opposed to cultural diffusion) described to date.

In addition to demography, a major focus of human population genetics studies over the past decade has been identifying regions of the genome that have been substrates of adaptive evolution. Several Symposium talks presented interesting examples of selection acting in humans. For example, Rasmus Nielsen (University of California Berkeley, USA) discussed his group's recent efforts in developing and applying new statistical tools for analyzing next-generation sequencing technology. Nielsen presented a maximum likelihood method for accurately reconstructing the site frequency spectrum from low-coverage data, a prerequisite for many downstream population genetics analyses. He also presented data on the application of these tools to exome data from individuals of Han Chinese and Tibetan ancestry, and the subsequent identification of the *EPAS1 *gene as a target of recent and strong selection in Tibetans which has played a major role in high-altitude adaptation.

Moreover, Michael Hammer (University of Arizona, Tucson, USA) presented data on levels of variation present on the X chromosome and autosomes to test hypotheses on the role that sexual selection has played in influencing patterns of human genomic diversity. Hammer showed that the expected ratio of X to autosomal variation was significantly different from the expected ratio of 0.75, and that the direction and magnitude of this ratio was strongly influenced by whether sequences analyzed were close to or far from genes. These results are difficult to reconcile by demography alone, and suggest that a complicated set of evolutionary forces, including selection, has influenced the ratio of X chromosome to autosomal diversity.

## Archaic genomes and introgression with modern humans

A particularly exciting series of talks focused on the genomes of extinct archaic human ancestors and current estimates on the amount of gene flow between archaic species and modern humans. Svante Pääbo (Max Planck Institute, Leipzig, Germany) summarized his group's work on obtaining whole-genome sequence data from Neandertals and Denisovans fossils. Pääbo estimates that approximately 2.5% of non-African genomes are derived from Neandertals and 5.8% of the genomes of individuals from Papua New Guinea and other regions of Melanesia are derived from Denisovans. These data suggest what Pääbo called a "leaky replacement" model of human origins in which modest amounts of gene flow occurred between anatomically modern humans and archaic ancestors, before these species were replaced.

Similarly, Jeff Wall (University of California San Francisco, USA) described a novel method for inferring archaic admixture, which he applied to publicly available whole-genome sequence data generated by Complete Genomics. Provocatively, he finds higher rates of introgression in Asians compared to Europeans. An advantage of Wall's method is that it does not require an archaic genome to infer introgression, and thus he was able to also test the hypothesis that contemporary African genomes have signatures of gene flow with archaic human ancestors. Strikingly, Wall indeed did find evidence of archaic admixture in African genomes, suggesting that modest amounts of gene flow were widespread throughout time and space during the evolution of anatomically modern humans.

## Very recent human history

In addition to talks focused on relatively ancient events, such as archaic admixture, two talks also addressed more recent aspects of human evolutionary history. Paradoxically, whereas a relatively small number of individuals can be used to infer older evolutionary events, accurate reconstruction of very recent demography requires much larger sample sizes. To this end, Joshua Akey (University of Washington, Seattle, USA) described patterns of protein-coding variation in 2,440 European and African-American exomes. Akey showed that the vast majority of protein-coding variation was exceptionally rare, novel and the result of recent accelerated population expansions. Importantly, the dramatic population expansions observed in the exome data were not detectable in smaller sample sizes, highlighting the utility of very large samples for illuminating recent evolutionary history.

In a similar vein, Mark Jobling (University of Leicester, UK) discussed the recent evolutionary history of Phoenicians, Roma, Anglo-Saxons and Vikings in Europe focusing on patterns of gene flow and population expansions. Jobling also discussed current challenges in interpreting patterns of human genetic variation, and emphasized the need for synergistic interactions between different fields, such as history, archaeology and linguistics in order to understand human history.

## Human phenotypic variation

There is also intense interest in correlating extant patterns of human genomic diversity with phenotypic differences among individuals. Obviously, this is crucial for understanding the heritable basis of human disease, but the genetic basis of non-disease related traits is also of fundamental importance. For example, in a creative project, Mark Shriver (Pennsylvania State University, University Park, USA) described his recent work in three-dimensional modeling of facial phenotypes. In particular, Shriver is performing admixture mapping in individuals of European and African ancestry to identify genetic variation associated with normal variation in facial phenotypes. Preliminary results have identified two genes with statistically significant effects on a variety of three-dimensional facial phenotypes, and these loci appear to be targets of recent and strong selection. Shriver also demonstrated that this work is likely to provide novel insights into the genetics of craniofacial dysmorphologies.

Also related to phenotypic variation, Gill Bejerano (Stanford University, Stanford, CA, USA) described an innovative and interdisciplinary approach for understanding the evolution of human specific regulatory mutations. Using both computational and experimental strategies, Bejerano has identified numerous functionally important and human-lineage specific regulatory mutations, which may contribute to uniquely human traits and susceptibility to disease. The study of regulatory variation is likely to be a fruitful approach for understanding human phenotypic variation, as it has long been hypothesized to be an important source of evolutionary change within and between species.

In short, this dynamic meeting highlighted some of the most intriguing and exciting recent developments in studying modern human genetic variation, and using patterns of variation present in contemporary populations to make inferences about our evolutionary past. The confluence of next-generation sequencing technology, novel statistical and computational tools, and the pursuit of interdisciplinary studies suggest that we can expect an even more exciting future in charting our past.

## Abbreviations

CNVs: Copy number variants.

## Competing interests

The author declares that they have no competing interests.

